# Investigation of factors responsible for cell line cytoplasmic expression differences

**DOI:** 10.1186/1471-2199-6-11

**Published:** 2005-05-11

**Authors:** Jonathan D Finn, Tabitha Wong, Pieter R Cullis

**Affiliations:** 1 Department of Biochemistry and Molecular Biology, University of British Columbia, Vancouver, B.C., Canada; 2 Inex Pharmaceuticals Corp., Burnaby, B.C., Canada

## Abstract

**Background:**

Previous work has described a novel cytoplasmic expression system that results in a 20-fold increase in the levels of gene expression over a standard CMV-based nuclear expression system, as compared with a 2–3 fold increase seen with previous similar systems. While this increase was seen with BHK and Neuro-2a cells, further studies revealed that some cell lines, such as COS-7, demonstrated relatively poor levels of cytoplasmic expression. The objective of this study was to determine what factors were responsible for the different expression levels between BHK (a high expressing cell line) and COS-7 (a low expressing cell line).

**Results:**

The main findings of this work are that the individual elements of the cytoplasmic expression system (such as the T7 RNAP gene and Internal Ribosome Entry Sequence) are functioning similarly in both cell types. Both cell types were found to have the same amount of cytosolic nuclease activity, and that the cells appeared to have differences in the intra-cellular processing of DNA -cationic lipid complexes.

**Conclusion:**

After exploring many factors, it was found that differences in the intra-cellular processing of the DNA-cationic lipid complex was the most probable factor responsible for the difference in cytoplasmic gene expression.

## Background

Previous work has described a novel cytoplasmic expression system that results in a 20-fold increase in the levels of gene expression over a standard CMV-based nuclear expression system [1], as compared with a 2–3 fold increase seen with previous similar systems [2]. While this increase was seen with BHK and Neuro-2a cells, further studies revealed that some cell lines, such as COS-7, demonstrated relatively poor levels of cytoplasmic expression.

Cytosolic nucleases have been discovered and characterized [3,4]. These proteins have found to be an important factor with respect to the cytosolic half-life of plasmid based vectors. These nucleases could be important to researchers using plasmid based delivery systems, especially cytoplasmic gene expression systems.

Internal Ribosome Entry Sequences (IRES) are elements that have been shown to drive expression of the second gene in a bi-cistronic mRNA transcript, as well as increase the translation of un-capped transcripts. IRES elements were first isolated from viral genomes, such as the encephalomyocarditis virus (EMCV), where they allow the translation of viral mRNA transcripts, made in the cytoplasm, and thus lacking the 5' cap structure essential for mRNA translation [5]. Various viral IRES elements, such as the EMCV, FMDV (foot and mouth disease virus), and other picornavirus based IRES elements share similar features. All are approx 450 bp long and share a conserved secondary structure, as well as a pyrimidine-rich tract that starts ~25 bp before the 3' end of the IRES [6-8]. The secondary structure is hypothesized to allow for proper alignment of ribosome subunits and other co-factors necessary for translation.

The objective of this study was to determine what factors were responsible for the different expression levels between BHK (a high expressing cell line) and COS-7 (a low expressing cell line). After investigating numerous factors, including the Internal Ribosome Entry Sequence (IRES) element, T7 RNAP activity, cytosolic nuclease levels and intra-cellular processing, it was found that differences in the intra-cellular processing of the DNA-cationic lipid complex was the most probable factor responsible for the difference in gene expression.

## Results

### Autogene transfection in COS-7 cells results in lower transgene expression than in BHK cells

**Figure 1:**
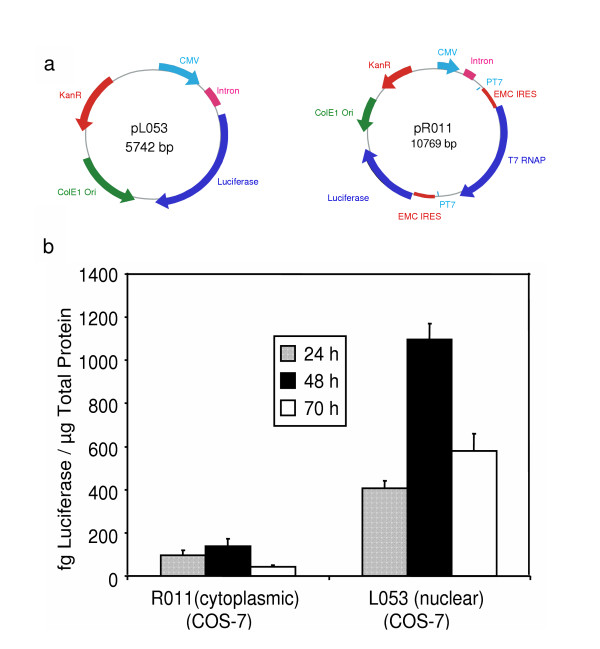
Autogene expression in COS-7 cells A) Diagrams of plasmids used. B) Comparison of cytoplasmic (R011) and nuclear (L053) expression plasmids in COS-7 cells. COS-7 cells were transfected with 0.75 μg/well of plasmid. Equimolar amounts of plasmids were added, and the total mass of DNA per transfection was kept equal by adding an unrelated plasmid (pPUC19). Transfections and luciferase assays were performed as described in Materials and Methods. Error bars indicate standard error.

In previous studies, it was found that the enhanced dual promoter autogene system demonstrated transgene expression levels that were 15–20 times higher than the equivalent nuclear control [1]. This was demonstrated for both BHK and Neuro-2a cells. However, when the system was tested in the COS-7 cell line, the autogene system resulted in levels of gene expression that were lower than the nuclear control (Figure 1). It had been hypothesized that the autogene system should produce high levels of gene expression in any transfected cell. Possible factors that would affect autogene expression levels including IRES function, T7 RNAP activity, cytosolic nuclease levels, and intra-cellular processing differences were therefore examined.

### EMCV IRES is functional in COS-7 cells

**Figure 2:**
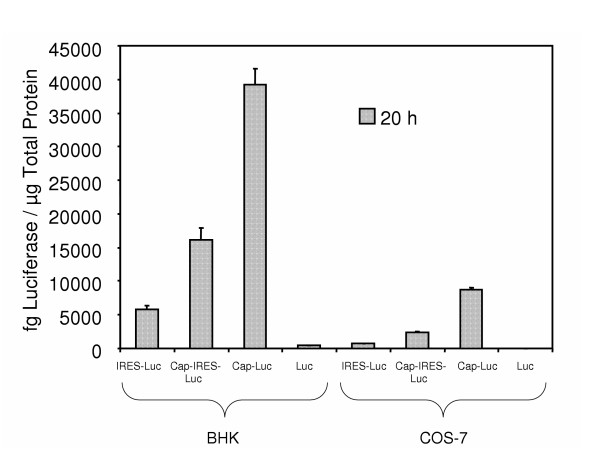
Transfection of BHK or COS-7 cells with various mRNA species. Cells were transfected with 0.75 μg/well of mRNA. Transfections and luciferase assays were performed as described in Materials and Methods. Error bars indicate standard error.

The first parameter that was tested was whether or not the EMCV IRES was functional in COS-7 cells. In order to determine this, mRNA was synthesized *in vitro *and transfected directly into either BHK or COS-7 cells. As is observed in Figure 2, the pattern of gene expression is similar for both cell lines. This indicates that while the IRES containing transcripts are not translated as efficiently as capped transcripts [9], the EMCV IRES has the same relative expression pattern in both of the cell types, indicating that differences in IRES function are most likely not the reason for the poor expression levels in COS-7 cells. It is interesting to note that the while the pattern of gene expression is similar, the absolute levels of gene expression are much lower in the COS-7 cells than the BHK cells. While it is known that cell division is an important factor for plasmid transfection, the BHK and COS-7 cells were found to have similar doubling times (data not shown), ruling this out as a factor responsible for the low expression levels in the COS-7 cells. Another reason could be because COS-7 cells are transfected (nucleic acid taken up and delivered into the cytoplasm or nucleus and subsequently expressed) less efficiently than BHK cells, or the intracellular processing of the nucleic acid-lipid complexes are different in the two cell lines, with the BHK cells having a greater amount of intact nucleic acid being released into the cytoplasm than the COS-7 cells. This possible difference in nucleic acid:cationic lipid complex processing could be responsible for the low levels of cytoplasmic autogene expression. It should be noted that we have used both BHK and COS-7 cells extensively and have determined that both cell lines are transfected with similar efficiency (~20%, data not shown), and that this efficiency is not dependent on the plasmid used.

### T7 RNAP is functional in COS-7 cells

**Figure 3:**
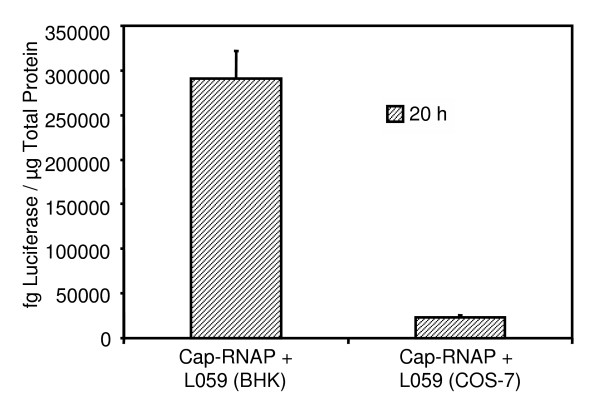
T7 RNAP is functional in COS-7 cells. Transfection of BHK or COS-7 cells with 0.75 μg of capped T7-RNAP mRNA for 24 hours, followed by a transfection with 0.75 μg of a luciferase reporter construct (L059) mRNA species. Cells were harvested 20 h post plasmid transfection. Transfections and luciferase assays were performed as described in Materials and Methods. Error bars indicate standard error. Note that in the absence of T7 RNAP (no pre-transfection) no luciferase was detected.

The next parameter that was examined was whether the T7 RNA polymerase (that is the basis of the cytoplasmic expression system) is active in COS-7 cells. Therefore, *in vitro *synthesized T7 RNAP mRNA was transfected directly into BHK or COS-7 cells. Twenty-four hours later cells were transfected with a luciferase reporter plasmid that would only express luciferase if functional T7 RNAP was being produced in those cells. As can be seen in Figure 3, while the levels of luciferase expression were lower in the COS-7 cells, these lower levels are consistent with the overall lower expression levels also seen in Figure 2. Note that in the absence of T7 RNAP pretreatment, no luciferase was detected. This data demonstrates that the T7 RNAP was indeed functional in the COS-7 cells. Therefore, these results indicate that the activity of the T7 RNAP gene was most likely not the reason for the poor expression levels.

### Effect of T7 RNAP mRNA pretreatment on autogene expression

**Figure 4:**
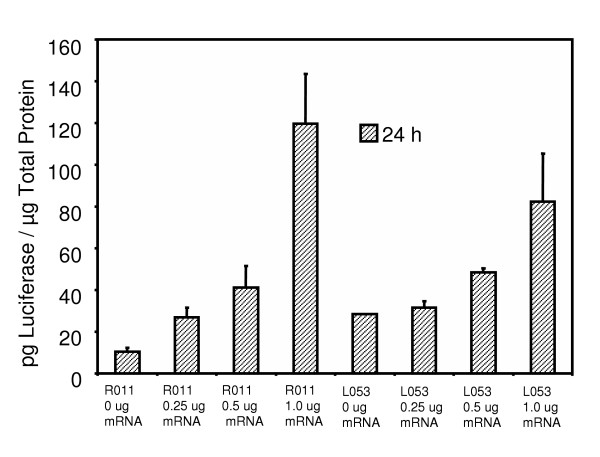
Pretreatment of COS-7 cells with T7 RNAP mRNA. Transfection of COS-7 cells with 0.75 μg of capped T7-RNAP mRNA for 24 hours, followed by a transfection with 0.75 μg of R011 (cytoplasmic) or L053 (nuclear) expression plasmids. Cells were harvested 24 h post plasmid transfection. Transfections and luciferase assays were performed as described in Materials and Methods. Error bars indicate standard error

Since it appeared as if all of the individual components of the autogene expression system were functioning in the COS-7 cells, it was suggested that perhaps the poor expression levels were due to temporal constraints. The cytoplasmic expression system is dependent on the initial source of T7 RNAP coming from the nucleus via the CMV promoter, which is then able to 'trigger' the cytoplasmic system. Perhaps by the time the first T7 RNAP proteins were produced via the nuclear transcripts (at least 8–12 hours post transfection), cytosolic nucleases had degraded the cytoplasmic plasmid, therefore leaving no substrate for the T7 RNAP. In order to test this hypothesis, BHK or COS-7 cells were transfected with T7 RNAP mRNA. Twenty-four hours post mRNA transfection cells were transfected with a plasmid encoding either the cytoplasmic expression system (R011) or the nuclear control (L053). As is apparent in Figure 4, the pretreatment of the COS-7 cells led to a 10 fold increase in autogene based expression that was not seen with the nuclear control. It was also observed that only about a 2–3 fold increase in expression was seen with the BHK cells (data not shown). This result appeared to support the hypothesis that timing was a major factor. If the cells were already primed with T7 RNAP protein prior to autogene expression, a substantial increase in gene expression was seen. These results suggest that certain factors affecting the amount of free cytoplasmic plasmid (such as cytoplasmic nuclease levels or the intracellular processing of complexes) are playing an important role in autogene expression differences between the BHK and COS-7 cells.

### BHK and COS-7 cells have similar levels of cytosolic nucleases

**Figure 5:**
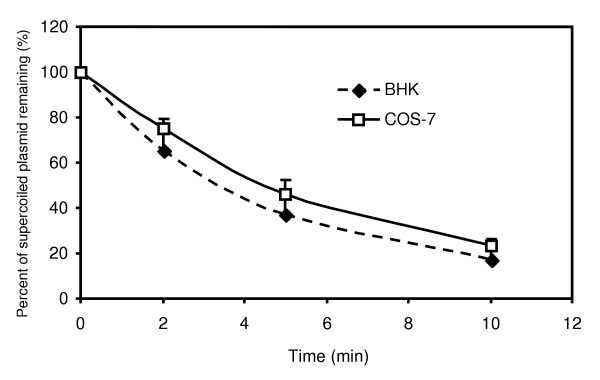
Cytosolic nuclease assays of BHK and COS-7 cytoplasmic extracts. 200 ng/μg plasmid DNA was incubated and aliquots were taken at time points indicated. No significant difference in nuclease activity between the BHK and COS-7 cytoplasmic extracts was observed.

In order to understand the difference between the high expressing (BHK) and low expressing (COS-7) cell lines, it was hypothesized that perhaps the BHK cells had lower levels of cytosolic nuclease, therefore more plasmid survived in the cytoplasm during the 8–12 h lag period before T7 RNAP was produced from the nuclear mRNA. Therefore, cytoplasmic extracts of both cell lines were obtained and subjected to nuclease assays. As can be seen in Figure 5, it appears as if the BHK cells did not have lower levels of cytoplasmic nuclease activity than the COS-7 cells, and may even appear to have slightly higher nuclease activity. Therefore, it appears as if the levels of cytosolic nuclease are not a major determining factor with respect to autogene based cytoplasmic expression.

### Intracellular processing of DNA is different in BHK and COS-7 cells

**Figure 6:**
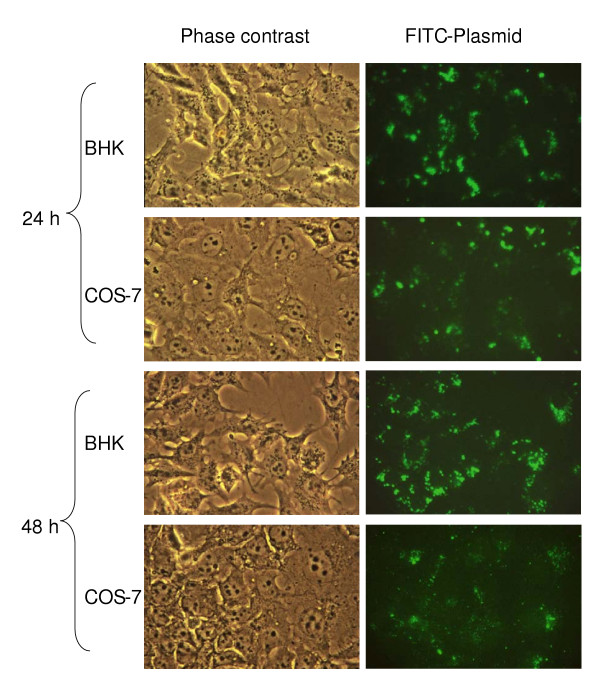
Intracellular processing of complexes. Transfection of BHK or COS-7 cells with FITC labeled R011. Cells were transfected with 0.75 μg of FITC-R011. Cells were fixed at time points indicated and subjected to phase contrast and fluorescence microscopy.

While it had been determined that the low levels of autogene expression in COS-7 cells was not due to increased levels of cytosolic nucleases, it could occur due to differences in the intracellular processing of DNA-lipid complexes between the two cell lines. In order to investigate this, plasmid DNA was labeled with a fluorescent label, transfected into both BHK or COS-7 cells, and then florescence microscopy was performed at various time points. As can be seen in Figure 6, while there appears to be a similar level of plasmid taken up into both cell lines at early time points (24 h), at later time points (48 h), the BHK cells seem to have accumulated a significant amount of plasmid in punctate, perinuclear structures, while the COS-7 cells have lost a significant amount of their fluorescence. Thus, it appears as if the BHK cells contain a pool of plasmid DNA for a longer period than the COS-7 cells. This perinuclear pattern seen with the BHK cell is characteristic of DNA-lipid complexes, and previous work [10] has demonstrated that these structures are not associated with lysosomes, and appear to be large structures, presumably formed when multiple endosomes have fused into larger vesicles. These studies clearly show that there is a difference in the intracellular processing of the DNA-lipid complexes between the BHK and COS-7 cells, and this is most likely the reason behind the lower levels of cytoplasmic gene expression seen with the COS-7 cells.

## Discussion

While previous work from our lab demonstrated a 15–20 fold increase in levels of gene expression using an autogene based, cytoplasmic expression system [1] in BHK and Neuro-2a cell lines, recent studies showed that the levels of cytoplasmic expression were significantly lower in some other cell lines, such as COS-7. In the present work, an attempt is made to determine what the reason for the difference was. Here various factors are discussed, including the activity of the various components of the autogene system in BHK and COS-7 cells, the finding that pretreating the COS-7 cells with T7 RNAP led to an increase in expression, and that the differences in expression between the two cell lines is most likely due to differences in intracellular processing of the DNA-lipid complexes.

Other studies have shown that IRES function is dependent on trans-acting cellular factors [11]. The pyrimidine tract binding protein (PTB) binds to the EMCV IRES [12], and functions by maintaining the IRES secondary structure, thereby facilitating translation through the IRES [13]. The La antigen has also been demonstrated to bind to the IRES [11,14]. There is also evidence for other proteins binding to various regions of IRES elements [15] that have been reported to have an effect on the IRES function. Therefore, it was thought that perhaps one of the differences between the BHK and COS-7 cells was the presence and/or abundance of various trans-acting cellular factors. However, transfection with various mRNA transcripts showed an identical pattern to that of the BHK cells, indicating that the EMCV IRES was functioning similarly in both of the cell lines, ruling out the IRES as the reason for the expression level differences.

It was then suggested that perhaps the T7 RNAP protein was not active in the COS-7 cells. This would also lead to poor expression levels. Transfecting the cells with capped mRNA encoding T7 RNAP, and then following 24 h later with a plasmid that will only express luciferase in the presence of T7 RNAP demonstrated that this was not the case. Even though the levels of expression were much lower than in BHK cells, a substantial amount of protein was expressed. It appears as if the COS-7 cells in general may not be very amenable to mRNA transfection, as both the transfections with the luciferase encoding mRNA transcripts (Figure 2) as well as the T7 RNAP encoding transcripts (Figure 3) gave much lower expression levels in the COS-7 cells than the BHK cells. This may be indicative of some difference in intracellular processing of the nucleic acid-lipid complex, allowing for less cytoplasmic delivery of mRNA.

As it appeared that the individual components of the autogene system were functioning, it was thought that perhaps the difference was due to a matter of timing. It is known that it takes at least 8–12 h post transfection in order to detect any expression from the nucleus, with expression usually peaking at around 24–48 h post-transfection. Perhaps in this time the majority of cytoplasmic plasmid in the COS-7 cells was degraded by cytosolic nucleases. By priming the cells with T7 RNAP (via pre-transfection of cells with T7 RNAP mRNA) prior to autogene transfection, a 10 fold increase in autogene expression was observed, as opposed to about a 2 fold increase with the nuclear control (most likely due to non-specific effects associated with increased lipid or nucleic acid delivery). Thus it was clear that the lag time between transfection and T7 RNAP expression from the nucleus was playing a role in the poor expression levels in COS-7 cells.

In order to further understand this phenomenon, it was proposed that the COS-7 cells had higher levels of a cytosolic nuclease, and this was degrading the cytoplasmic plasmid, decreasing the amount of intact plasmid left for cytoplasmic expression. However, nuclease assays on cytoplasmic extracts from BHK or COS-7 cells showed that in fact, the COS-7 cytoplasmic extracts had less nuclease activity than the BHK cells. Thus, increased levels of cytosolic nucleases in the COS-7 cells was not the reason for the decreased expression.

The observation that the mRNA transfections gave much lower levels of expression in COS-7 cells than BHK cells hinted at the fact that perhaps there were differences in the intracellular processing of the DNA-lipid complexes, with the COS-7 cells allowing for less free plasmid to be released into the cytoplasm.

By labeling the plasmid with a fluorescent label (FITC) and looking at the internal distribution of plasmid in both BHK and COS-7 cells over time, it was determined that there was a qualitative difference in not only the localization of the labeled plasmid, but also in the amount of plasmid. It appears as if in the COS-7 cells, the plasmids are degraded more quickly, shown by the less intense overall fluorescence as well as the diffuse green staining throughout the cell, indicative of plasmid degradation. This could be due to either a greater number of complexes being transported to lysosomes in COS-7 cells, or that the complexes are being released from the endosomes faster, and therefore being degraded by cytosolic nucleases before the cytoplasmic expression system can be triggered. In contrast, the BHK cells contained larger pools of plasmid DNA in discrete, non-lysosomal, perinuclear structures, even after 48 h. It is hypothesized that perhaps plasmid is being released into the cytoplasm over time, thereby allowing more free cytoplasmic plasmid to be available for cytoplasmic transcription at later time points.

## Conclusion

In conclusion, it has been demonstrated that cell line differences in cytoplasmic expression (when compared to nuclear expression) are most likely due to differences in the intra-cellular processing of the nucleic acid-cationic lipid complexes, as opposed to differences in how the individual components (IRES, RNAP, etc.) function.

## Methods

### Plasmids

Plasmid R011 is a bi-cistronic plasmid consisting of a basic autogene cassette (containing the T7, T3 and SP6 RNAP promoters) driven by a CMV promoter and intron, as well as a downstream *Photinus pyralis *luciferase reporter gene cassette (for construction details see Finn et al, 2004). L053 consists of the CMV promoter (with intron) from NGVL3 and the *Photinus pyralis *luciferase gene. L059 consists of a pTRI-Amp (Ambion) backbone with EMCV IRES, *Photinus pyralis *luciferase and beta-globin poly-adenylation site derived from EMC-Luc, a gift from Dr. Jon Wolff (Waisman Center, Wisconsin). PT7-Luc (Promega) consists of the *Photinus pyralis *luciferase gene driven by a T7 RNAP promoter. L080 consists of PT7-Luc with the Luciferase gene driven by both the T7 and the SP6 promoter.

### mRNA synthesis

mRNA synthesis was performed using a MEGAscript high yield transcription kit (Ambion). For all transcripts used for *in vitro *transfection, plasmid template (L059 for IRES-Luc and Cap-IRES-Luc mRNA; L080 for Cap-Luc mRNA; PT7-Luc for Luc mRNA) was linearized using *EcoR *1 and 1 μg of each plasmid was used as per manufacturers protocols. mRNA was recovered by the LiCl procedure as outlined in the manufacturer's protocol. After LiCl precipitation, the RNA pellet was washed 3 × with 70% ethanol to completely remove any unincorporated NTPs.

### Transfections

Lipoplexes were formed by mixing plasmid DNA with large unilamellar vesicles (LUVs) composed of equimolar amounts of DOPE:DODAC (50:50) on ice and incubated for 20 min prior to use. All transfections were performed at a cationic lipid to plasmid DNA charge ratio of 3:1. Lipoplexes were diluted with serum-containing media before addition directly to cell medium. BHK or COS-7 cells were plated at 30,000 cells per well in 24-well plates. The total mass of plasmid added was identical in all transfections. Equimolar transfections using experimental plasmids of different sizes were achieved through the addition of an empty vector (pPUC19) to normalize the total mass of DNA in each transfection. For mRNA transfections, TransMessenger reagent (Qiagen) was used to transfect differing amounts of various mRNA transcripts into BHK cells (30,000 cells per well in 24-well plates). An RNA to TransMessenger reagent ratio of 1:4 was used for all transfections. Transfections were performed as per manufacturer's instructions. Three hours post transfection the medium was removed and replaced with fresh serum containing medium. Cells were harvested and subjected to luciferase assays at time points indicated. For mRNA pretreatment studies, cells were transfected with mRNA as described above, followed by a DNA transfection 24 h later. All transfections were performed in triplicate. Data is presented as mean values +/- standard error.

### Luciferase and BCA assays

Cells were washed twice with 1 mL PBS followed by the addition of 0.2 mL lysis buffer (PBS with 0.1 % Triton X-100) before being stored at -70°C. Cells were thawed and 5–20 μl of sample were assayed in duplicate on a 96-well plate. Samples were assayed using a Berthhold Centro LB960 Microplate Luminometer and Luciferase Assay System (Promega). Standard luciferase assays were performed and transfection data is reported as mass quantities of luciferase protein using a standard curve obtained from serial 10-fold dilutions of a 20 mg/mL *Photinus pyralis *luciferase standard (Promega). Total protein was quantified using a Pierce BCA assay kit as per manufacturer's instructions.

### Cytoplasmic extract preparation

Cytoplasmic extracts were prepared using a hypotonic buffer solution. Cells (2 million) were washed with PBS (2 × 1 mL) before a 15 min incubation on ice in 500 μl hypotonic extraction buffer (10 mM Hepes-KOH pH 7.9, 1.5 mM MgCl2, 10 mM KCl, 0.5 mM DTT, 0.2 mM PMSF, 0.6% NP-40). The samples were centrifuged at 13,000 × g for 3 min at 4°C and the supernatant was collected and saved as the cytoplasmic fraction. Protein levels were quantitated using a Pierce BCA assay (as described above) after exchanging the hypotonic extraction buffer with PBS using a centrifugal filter device with a MW cut off of 3000.

### Nuclease assay procedure

Various amounts of cytoplasmic extract were incubated with plasmid DNA (50–300 ng extract/μg plasmid DNA) at 37°C. At time points indicated, aliquots were removed and flash frozen in liquid nitrogen and stored at -70°C until all assays were completed. Samples were then thawed and run on a 0.8% agarose gel and bands were visualized using ethidium bromide. Nuclease activity defined by the proportion of intact supercoiled plasmid left over time.

### Plasmid labeling

R011 (cytoplasmic expression plasmid) was labeled using a *Label *IT Tracker Fluorescein Kit from Mirus Corporation following manufacturers instructions. A ratio of 1 μl *Label *IT Tracker Reagent per μg of plasmid DNA was used. Transfections were performed as previously described.

### Fluorescence microscopy

At time points indicated, cells were washed 3 × with room temperature PBS before fixing for 5 min with 2% paraformaldeyde. Cells were further washed with 2 × 1 mL PBS before being treated with Prolong Antifade (Molecular Probes) and mounted. Pictures were taken using a Zeiss Axiovert S100 fluorescence microscope. In all cases, exposure times were kept constant to allow for ease of comparison of samples.

## Authors' contributions

JF completed all of the experimental design and carried out 90% of the experimental work and wrote 80% of the manuscript. TW completed all of the tissue culture associated with the work. PC has written 20% of the manuscript and was responsible for critically revising it and giving the final verision approval for publication
